# Functionally specified protein signatures distinctive for each of the different blue copper proteins

**DOI:** 10.1186/1471-2105-5-127

**Published:** 2004-09-09

**Authors:** Anuradha Vivekanandan Giri, Sharmila Anishetty, Pennathur Gautam

**Affiliations:** 1 Centre for Biotechnology, Anna University, Chennai 600 025, India; 2 AU-KBC Research Centre, Anna University, Chennai 600 044, India

**Keywords:** Blue copper proteins (BCP), ClustalW, Protein signatures, Peptide

## Abstract

**Background:**

Proteins having similar functions from different sources can be identified by the occurrence in their sequences, a conserved cluster of amino acids referred to as pattern, motif, signature or fingerprint. The wide usage of protein sequence analysis in par with the growth of databases signifies the importance of using patterns or signatures to retrieve out related sequences. Blue copper proteins are found in the electron transport chain of prokaryotes and eukaryotes. The signatures already existing in the databases like the type 1 copper blue, multiple copper oxidase, cyt b/b6, photosystem 1 psaA&B, psaG&K, and reiske iron sulphur protein are not specified signatures for blue copper proteins as the name itself suggests. Most profile and motif databases strive to classify protein sequences into a broad spectrum of protein families. This work describes the signatures designed based on the copper metal binding motifs in blue copper proteins. The common feature in all blue copper proteins is a trigonal planar arrangement of two nitrogen ligands [each from histidine] and one sulphur containing thiolate ligand [from cysteine], with strong interactions between the copper center and these ligands.

**Results:**

Sequences that share such conserved motifs are crucial to the structure or function of the protein and this could provide a signature of family membership. The blue copper proteins chosen for the study were plantacyanin, plastocyanin, cucumber basic protein, stellacyanin, dicyanin, umecyanin, uclacyanin, cusacyanin, rusticyanin, sulfocyanin, halocyanin, azurin, pseudoazurin, amicyanin and nitrite reductase which were identified in both eukaryotes and prokaryotes. ClustalW analysis of the protein sequences of each of the blue copper proteins was the basis for designing protein signatures or peptides. The protein signatures and peptides identified in this study were designed involving the active site region involving the amino acids bound to the copper atom. It was highly specific for each kind of blue copper protein and the false picks were minimized. The set of signatures designed specifically for the BCP's was entirely different from the existing broad spectrum signatures as mentioned in the background section.

**Conclusions:**

These signatures can be very useful for the annotation of uncharacterized proteins and highly specific to retrieve blue copper protein sequences of interest from the non redundant databases containing a large deposition of protein sequences.

## Background

Most proteins can be grouped, on the basis of similarities in their amino acid sequences, into a limited number of protein families. Proteins or protein domains that belong to a particular family usually share functional attributes and are derived from a common ancestor. Highly conserved sequences in protein families are generally important for the function of a protein and/or for the maintenance of its 3-dimensional structure. Within the last decade, the sensitivity of sequence searching techniques has been improved by profile or motif-based analysis, which uses information derived from multiple sequence alignments to construct and search for sequence patterns [1-4]. By studying constant and variable properties of such groups, a signature for a protein family or domain can be derived which distinguishes its members from all other unrelated proteins. The problem of fast exact and approximate searching for a pattern that contains classes of characters and bounded size gaps (CBG) in a text has a wide range of applications, among which a very important one is protein pattern matching [5]. Unlike single-sequence similarity, a profile or motif can exploit additional information, such as the position and identity of residues that are conserved throughout the family, as well as variable insertion and deletion probabilities [6]. These signatures can be used to assign a newly sequenced protein to a specific family to formulate hypotheses about its function.

By doing a keyword search, the protein sequences mined out from different databases is highly varied owing to different levels of redundancy. This could be due to the different strengths and weaknesses underlying the analysis algorithms used in different databases. The usage of the broad range signatures existing in databases, for the retrieval of blue copper proteins like the type 1 copper blue, multiple copper oxidase, cyt b/b6, photosystem 1 psaA&B, psaG&K, and reiske iron sulphur protein brings out different kinds of copper proteins and a lot more of unrelated proteins. A search once again becomes necessary for sorting out the required blue copper proteins. The usage of pattern database would be more selective as it can identify family members based on the conserved functional region patterns. Keeping these broad spectrum signatures in mind, more specific and targeted protein signatures for each of the blue copper proteins was designed. The diagnostic success of these specified signatures over the wide range signatures mentioned lies in the number of true positives picked over the minimal or nil false positives picked from the non redundant databases.

**Figure 1:**
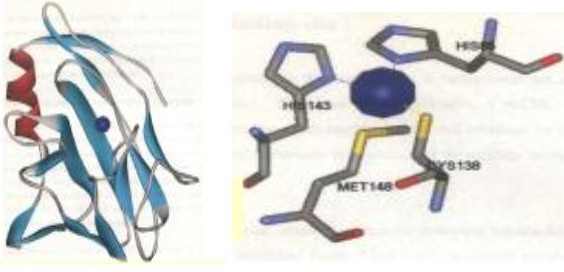
a) Rusticyanin bound to the copper atom as indicated by the blue colored ball. b) Copper binding active site of rusticyanin showing the four active site residues: Histidine at 85^th ^position, Histidine at 143^rd ^position, Cysteine at 138^th ^position and Methionine at 148^th ^position

Blue copper proteins, which are also known as cupredoxins, are small, soluble proteins (10 – 14 kDa) whose active site contains a type 1-copper [7]. All these type 1 blue copper proteins possess an eight stranded Greek key beta barrel or beta sandwich fold and have a highly conserved active site architecture. The type 1 blue copper proteins exert their function by shuttling electrons from a protein acting as an electron donor, to another acting as an electron acceptor in various biological systems such as bacterial and plant photosynthesis [8,9]. During the electron transfer process, the copper ion changes from a diamagnetic Cu(I) to a paramagnetic Cu(II), oxidation state [10]. The coordination of the copper is determined by the conformation of its three closest ligands, two histidine nitrogens and a cysteine sulfur and of a fourth more distant ligand a methionine sulphur [11]. The coordination sphere of copper ions in blue copper protein rusticyanin is shown for example in Figure 1. Type 1 copper sites are characterized by an intense blue color due to copper bound to thiolate [12]. An absorption is seen at 600 nm and gives rise to an unusual EPR signal, arising from asymmetrical copper site. Most of the cupredoxins have similar redox potentials ranging from 260 to 375 mV and function at pH values ranging from 6 to 8 [9]. Rusticyanin is an exception in having a very high redox potential of 680 mV [13].

The use of active site patterns or signatures is very rapidly becoming one of the essential tools of sequence analysis [14,15]. Although there is an appreciable amount of divergence in the sequences of the different blue copper proteins, the copper ligand sites are conserved. Direct application of the functionally specified signatures in databases, would help in quick retrieval of protein sequences related to that signature. The protein sequences thus retrieved were found to be highly specific to a particular blue copper protein. These signatures being highly specific allow the efficient mining out of uncharacterized proteins from the vast sequences deposited in different databases.

## Results

### Differentiation of blue copper proteins based on source of origin and active site tabulation

The eukaryotic blue copper proteins chosen for the study were plantacyanin, plastocyanin, cucumber basic protein, stellacyanin, umecyanin, uclacyanin, and cusacyanin. The prokaryotic blue copper proteins were rusticyanin, sulfocyanin, halocyanin, azurin, pseudoazurin, auracyanin, amicyanin and blue nitrite reductase. Plastocyanins are found both in eukaryotes and prokaryotes. Table 1 and 2 describe the active site functional region for each of the blue copper proteins mentioned above. The active site functional region indicates the aminoacids in the respective blue copper proteins bound to the copper atom. For example in plantacyanin with the protein data bank id 1F56, histidine at the 34^th ^position, cysteine at the 74^th ^position, histidine at 79^th ^position and methionine at the 74^th ^position are bound to the copper atom involved in electron transport chain.

### Keyword search for the specified blue copper proteins in different databases

The number of sequences retrieved for a protein from different databases by keyword search are tabulated in Table 3. As seen from Table 3, a keyword search is no longer effective and precise in retrieving sequences of a particular kind. If still used, it is only a time consuming process, as the particular protein of interest has to be filtered from the retrieved sequences once again. For example, a varied response of data output is seen on a keyword search for plastocyanin. The sequences retrieved from each of the database in terms of number of protein sequences is 901 sequences in NCBI, 41 sequences in SwissProt, 10 sequences in TrEMBL, 350 sequences in Protein Information Resource, 375 sequences in EMBL, and 41 sequences in PDB.

### A search for the existing signatures for the blue copper proteins

The signatures already available for each of the blue copper proteins retrieved from the Prosite motif database are listed in Table 4. The number of protein sequences retrieved in response to the input of the already existing signatures for blue copper proteins in the PIR nREF database is shown in Table 5. An overview of the results in Table 4 indicates that most of the blue copper proteins have a type 1 blue copper signature with an id PS00196. The multiple copper oxidase signature present in rusticyanin as shown in Table 4 with id PS00079 and PS00080 retrieves out 799 and 366 sequences respectively as shown in Table 5. The existing rusticyanin sequences are very few in actual number and hence a secondary search becomes necessary. Even if the signature has annotated an unknown protein such as rusticyanin, it has to be searched amongst the 779 and 366 sequences retrieved. From Table 4 it is seen that plastocyanin has cyt b-heme (PS00192), cyt b QO(PS00193), photosystem1 PSAAB (PS00419), photosystem1 PSAGK(PS01026), Reiske 1 (PS00199) and Reiske 11(PS00200) as the signatures. As the names of the signatures suggest they are highly broad spectrum. The number of sequences picked out by these signatures as shown in Table 5 clearly indicates that most of these signatures are picking a lot more of other sequences other than plastocyanin and some of the signatures are missing out some plastocyanin sequences.

### Designing functional protein signatures for the different blue copper proteins

As shown in 'Appendix 1 [see Additional file 1]' the newly designed signatures and peptides based on the ClustalW results for plastocyanin sequences of both eukaryotic and prokaryotic origin are shown in Table 6. The same procedure as shown in Appendix 1 [see Additional file 1] was followed for the other blue copper proteins and the newly designed signatures and peptides based on the ClustalW results are shown in Table 6. The new signatures and peptides thus designed for the different blue copper proteins, picked out highly specific sequences from the PIR nREF database consisting of sequences from the PIR [282,987 sequences], SwissProt [146,720 sequences], TrEMBL [1,069,649 sequences], GenPept [1,724,186 sequences], Ref Seq [756,736 sequences] and PDB [24,863 sequences]. The number of sequences retrieved based on the new signature for each of the blue copper protein is shown in Table 6.

## Discussion

The members of a protein family can be identified by collecting the matching sequences to profile or motif databases. Protein signatures are sequence motifs diagnostic to a protein family indicating function. Signatures are matched to protein sequences in the non redundant databases and is scored using a dynamic programming algorithm which permits permeability in gap distance and residue type [16]. Generating a signature involves identifying residues in a protein sequence that imparts functional properties to the protein. Protein signatures are efficient miners of related protein sequences having the same functional residues, which belong to the same class of proteins from the abundant sequences present in the non redundant databases. All the copper ions in the living cells are protein bound, as it is toxic in its free form. In most copper proteins, the copper ion having the ability to change valence state is mainly involved in catalysis of biological process, or the transport of electrons different proteins in a cell. Blue copper proteins also known as cupredoxins, have a type I copper site. They possess a single copper functional domain. The coordination of copper in most of the blue copper proteins is determined by the conformation of its three closest ligands, two histidine nitrogens and a cysteine sulfur and of a fourth more distant ligand a methionine sulfur [11]. In the case of auracyanin, stellacyanin and umecyanin the methionine is substituted by a glutamine residue, which binds as the fourth ligand to the copper atom.

By doing a keyword search, we get varied results from the different databases as indicated in Table 3 owing to different levels of redundancy. On using a functionally related protein signature only relevant related sequences are picked out from the non redundant database as seen from Table 6. Thus protein signatures can play a great role in extracting out highly related sequences from different databases than keyword searches. The signatures already available for the blue copper proteins like the Cyt b/b6, Photosystem 1 PSAGK, Rieske Iron Sulfur protein, and type I copper blue signatures are broad spectrum signatures. PS00196 a type I blue copper signature which is an already existing signature, when fed in the PIR nREF database has picked out 589 sequences as indicated in the result in Table 5. We have also ensured that the active site region involving amino acids bound to the copper atom is present in all our signatures. Protein signatures designed taking into account the active site region will be very efficient for annotation of uncharacterized proteins. In one study the authors have used metal binding patterns of metalloproteins present in Protein Data Bank to search gene banks for new metalloproteins [17].

The protein signatures in a way can be compared to primers used for amplification. The more specific and concise a primer, the more specific is the amplification, similarly more specific the protein signature more significant are the picks from the non redundant databases. Specific signatures in a way reduce the time taken to pool related sequences from the abundantly available sequences from the non redundant databases. For example as shown in Table 4 the Type 1 blue copper signature is present in plantacyanin, stellacyanin, umecyanin, cusacyanin, halocyanin, azurin, auracyanin and nitrite reductase amongst the sixteen different blue copper proteins. When this signature is used as a query in the Prosite database even if an unknown protein is annotated it can only be as a type 1 blue copper protein, but it cannot classify it as a particular blue copper protein. The newly designed signatures or peptides will help in classifying the uncharacterized protein to the exact subtype of blue copper proteins. In this study, we have assigned functional property based signatures, which have the amino acid residues binding to the copper atom. It may be concluded that we have been successful in designing functionally related protein signatures for the blue copper proteins.

## Conclusions

Signatures designed around the functionally important regions of a protein are valuable for annotation. In this study, specific signatures were designed around the active site regions of each of the blue copper proteins plantacyanin, plastocyanin, uclacyanin, stellacyanin, rusticyanin, sulfocyanin, amicyanin, halocyanin, pseudoazurin, azurin and nitrite reductase. These will be very useful for annotating uncharacterized proteins as blue copper proteins. Further, because of their high specificity to each subclass, they can be used in classifying the various subtypes of blue copper proteins.

## Methods

### Differentiation of blue copper proteins based on source of origin and active site tabulation

The blue copper proteins were distinguished based on the source of origin as prokaryotic and eukaryotic. The active site residues of the eukaryotic and prokaryotic blue copper proteins, which bind the copper metal atom, were identified from the Protein Data Bank and tabulated.

### Keyword search for the specified blue copper proteins in different databases

The name of each of the blue copper protein was given as a query in the keyword searches at NCBI, SwissProt, TrEMBL, PIR, EMBL and PDB databases to check for the number of sequences retrieved from each database and the results were tabulated.

### A search for the existing signatures for the blue copper proteins

The signatures already existing for each of these blue copper proteins were identified from the Prosite motif database and tabulated. These already existing signatures were used as query patterns in the PIR Motif/Peptide match and a search was made against the PIR-nREF database. The PIR-nREF database consists of sequences from the PIR [282,987 sequences], SwissProt [146,720 sequences], TrEMBL [1,069,649 sequences], GenPept [1,724,186 sequences], Ref Seq [756,736 sequences] and PDB [24,863 sequences]. The number of protein sequences matching the query (already existing signatures for the blue copper proteins) was retrieved and the query results were tabulated.

### Designing functional protein signatures for the different blue copper proteins

Each of the blue copper proteins from different sources were chosen from the different sequence databases and aligned using the multiple sequence alignment tool ClustalW. Conserved regions, which include the amino acids bound to the copper, reflecting the active site imparting a vital biological role of electron transport were chosen to design signatures. In regions showing 100% conserved sequences they were identified as conserved peptides. An example of how the functional protein signatures were designed is shown for plastocyanin in Appendix 1 [see Additional file 1]. The signatures and peptides were then submitted to the Protein Information Resource [PIR nREF] database for the protein pattern and peptide match and the results were tabulated.

## Authors' Contributions

PG, AVG and SA participated in the design and coordination of the study. AVG carried out the bioinformatics search, the designing of the signatures and drafted the manuscript. PG and SA read and approved the final manuscript.

## Supplementary Material

Additional File 1 Designing protein signatures: Illustrated example Plastocyanin. Plastocyanin sequences of eukaryotic and prokaryotic origin were retrieved from the PDB and SwissProt databases. The eukaryotic sequences were subjected to a ClustalW multiple sequence alignment. Signatures were designed based on the conserved pattern around the active site region [copper binding to four amino acids in plastocyanin]. The same procedure was adopted for plastocyanin sequences of prokaryotic origin. The newly designed signatures were used as queries in the Pattern/peptide match search at the PIR database [Protein Information Resource]. The numbers of plastocyanin sequences retrieved are tabulated in Table 6. The results were compared with the already existing signatures for plastocyanins and the number of sequences that these signatures picked up from the PIR database [data shown in Table 4 & 5]. Click here for file

## Figures and Tables

**Table 1:** Amino acids bound to the copper atom of the blue copper proteins of eukaryotic origin. Plastocyanins are also found in prokaryotes. The * indicates that crystal structures are as yet not available.

**S. NO:**	**PROTEIN NAME**	**METAL BINDING ACTIVE SITES**			
		
		**1**	**2**	**3**	**4**
1	PLANTACYANIN PDB: 1F56	34 HIS	74CYS	79 HIS	84 MET
2	PLASTOCYANIN PDB: 1AG6	37 HIS	84 CYS	87 HIS	92 MET
3	CUCUMBER BASIC PROTEIN PDB: 2CBP	39 HIS	79 CYS	84 HIS	89 MET
4	STELLACYANIN1JER	46 HIS	89 CYS	94 HIS	99 GLN
5	DICYANIN	*	*	*	*
6	UMECYANIN P42849	44 HIS	85 CYS	90 HIS	95 GLN
7	UCLACYANIN	*	*	*	*
8	CUSACYANIN P00303	39 HIS	79 CYS	84 HIS	89 MET

**Table 2:** Amino acids bound to the copper atom of the blue copper proteins of prokaryotic origin.

**S. NO:**	**PROTEIN NAME**	**METAL BINDING ACTIVE SITES**			
		
		**1**	**2**	**3**	**4**
1	RUSTICYANIN PDB: 1A8Z	85 HIS	138 CYS	143 HIS	148 MET
2	SULFOCYANIN Q53765	110 HIS	171 CYS	176 HIS	181 MET
3	HALOCYANIN P39442	110 HIS	148 CYS	151 HIS	156 MET
4	AZURIN PDB: 1DYZ	46 HIS	112 CYS	117 HIS	121 MET
5	PSEUDOAZURIN PDB: 1ADW	40 HIS	78 CYS	81 HIS	86 MET
6	AURACYANIN PDB: 1QHQ	57 HIS	122 CYS	127 HIS	132 GLN
7	AMICYANIN PDB: 1AAC	53 HIS	92 CYS	95 HIS	98 MET
8	NITRITE REDUCTASE PDB: 1AS6	95 HIS	136 CYS	145 HIS	150 MET

**Table 3:** Number of retrieved protein sequences from different databases on a keyword search

**S. NO:**	**PROTEIN**	**NCBI**	**SWISS PROT**	**TrEMBL**	**PIR**	**EMBL**	**PDB**
1	PLANTACYANIN	18	1	4	10	9	2
2	PLASTOCYANIN	988	41	22	463	397	40
3	CUCUMBER BASIC PROTEIN	6	1	*	*	4	1
4	STELLACYANIN	26	3	3	33	12	2
5	UMECYANIN	4	1	*	1	1	*
6	UCLACYANIN	23	1	5	9	9	*
7	CUSACYANIN	2	1	*	1	1	1
8	DICYANIN	1	*	1	1	1	*
9	RUSTICYANIN	52	2	8	22	25	7
10	SULFOCYANIN	14	1	5	6	6	*
11	HALOCYANIN	15	1	4	7	5	*
12	AZURIN	320	22	23	202	66	49
13	PSEUDOAZURIN	68	6	4	25	22	16
14	AURACYANIN	10	1	2	5	3	2
15	AMICYANIN	105	3	3	34	34	10
16	NITRITE REDUCTASE	1744	37	735	1027	1017	69

**Table 4:** Already existing signatures present in the Prosite motif database for the blue copper proteins

**S. NO:**	**PROTEIN**	**PDOCOO-NO:**	**PROSITE**	**SIGNATURE**
1	PLANTACYANIN	PDOC00174 TYPE-1 COPPER BLUE	1. PS00196	[GA]-x(0,2)-[YSA]-x(0,1)-[VFY]-x-[C]-x(1,2)-[PG]-x(0,1)-[H]-x(2,4)-[MQ]
2	PLASTOCYANIN	PDOC00171 Cyt b/b6	1. PS00192 – Cyt b Heme	[DENQ]-x(3)-[G]-[FYMWQ]-x-[LIVMF]-[R]-x(2)-H
			2. PS00193 – Cyt b QO	[P]-[DE]-[W]-[FY]-[LFY](2)
		PDOC00347 Photosystem 1 psaA and psaB	1. PS00419 – Photosystem 1 PSAAB	CDGPGRGGTC
		PDOC00786 Photosystem 1 psaG and psaK	1. PS01026 – Photosystem 1 PSAGK	[GT]-[F]-x-[LIVM]-x-[DEA]-x(2)-[GA]-x-[GTA]-[STA]-x-[GH]-x-[LIVM]-[GA]
		PDOC00177 Reiske-Iron Sulfur	1. PS00199 – Reiske 1	[C]-[TK]-[H]-[L]-[G]-[C]-[LIVST]
			2. PS00200 – Reiske 11	[C]-[P]-[C]-[H]-x-[GSA]
3	CUCUMBER BASIC PROTEIN	*	*	*
4	STELLACYANIN	PDOC00174 Type 1 Blue Copper	1. PS00196	[GA]-x(0,2)-[YSA]-x(0,1)-[VFY]-x-[C]-x(1,2)-[PG]-x(0,1)-[H]-x(2,4)-[MQ]
5	UMECYANIN	PDOC00174 Type 1 Blue Copper	1. PS00196	[GA]-x(0,2)-[YSA]-x(0,1)-[VFY]-x-[C]-x(1,2)-[PG]-x(0,1)-[H]-x(2,4)-[MQ]
6	UCLACYANIN	*	*	*
7	CUSACYANIN	PDOC00174 Type 1 Blue Copper	1. PS00196	[GA]-x(0,2)-[YSA]-x(0,1)-[VFY]-x-[C]-x(1,2)-[PG]-x(0,1)-[H]-x(2,4)-[MQ]
8	DICYANIN	*	*	*
9	RUSTICYANIN	PDOC00076 Multiple Copper Oxidase	1. PS00079 – Copper Oxidase	[G]-x-[FYW]-x(LIVMFYW)-x-[CST]-x(8)-[G]-[LM]-x(3)-[LIVMFYW]
			2. PS00080 – Multiple Copper Oxidase	[H]-[C]-[H]-x(3)-[H]-x(3)-[AG]-[LM]
10	SULFOCYANIN	*	*	*
11	HALOCYANIN	PDOC00174 Type 1 Blue Copper	1. PS00196	[GA]-x(0,2)-[YSA]-x(0,1)-[VFY]-x-[C]-x(1,2)-[PG]-x(0,1)-[H]-x(2,4)-[MQ]
12	AZURIN	PDOC00174 Type 1 Blue Copper	1. PS00196	[GA]-x(0,2)-[YSA]-x(0,1)-[VFY]-x-[C]-x(1,2)-[PG]-x(0,1)-[H]-x(2,4)-[MQ]
13	PSEUDOAZURIN	*	*	*
14	AURACYANIN	PDOC00174 Type 1 Blue Copper	1. PS00196	[GA]-x(0,2)-[YSA]-x(0,1)-[VFY]-x-[C]-x(1,2)-[PG]-x(0,1)-[H]-x(2,4)-[MQ]
15	AMICYANIN	*	*	*
16	NITRITE REDUCTASE	PDOC00174 Type 1 Blue Copper	PS00196	[GA]-x(0,2)-[YSA]-x(0,1)-[VFY]-x-[C]-x(1,2)-[PG]-x(0,1)-[H]-x(2,4)-[MQ]

**Table 5:** Number of protein sequences retrieved from the PIR nREF database for the already existing signatures for the blue copper proteins

**PROSITE ID**	**PROSITE PATTERN SEARCH RESULTS**
PS00079	779
PS00080	366
PS00192	18593
PS00193	13818
PS00196	589
PS00199	158
PS00200	284
PS00419	348
PS01026	3

**Table 6:** Newly designed protein signatures/peptides for the different blue copper proteins. The number of protein sequences retrieved from the PIR nREF database for each of the newly designed signatures.

**S. NO:**	**NAME OF THE PROTEIN**	**SIGNATURE /PEPTIDES**	**NO: OF RELATED PICKS**	**NO: OF FALSE PICKS**
1	PLANTACYANIN	[F]-[I]-[C]-[NST]-[F]-[PG]-x-[H]-[C]	10	NO
2	PLASTOCYANIN [eukaryotic source]	[C]-x-[P]-[H]-x-[GS]-[A]-[GN]-[M]	80	1
3	PLASTOCYANIN [prokaryotic source]	[Y]-[C]-x-[P]-[H]-[R]-[G]-[A]-[G]-[M]-[V]-[G]	21	NO
4	PLASTOCYANIN [prokaryotic source]	PHRGAGMVG	21	NO
5	STELLACYANIN	[H]-[C]-x-[NL]-[G]-[MQ]-[K]-[LV]-x-[VI]-[NRQ]-[V]	7	1
6	UCLACYANIN	[P]-[G]-x-[R]-[Y]-[F]-[I]-[C]-[G]	3	NO
7	UCLACYANIN	RYFICG	11	NO
8	RUSTICYANIN	[G]-[M]-[YF]-[G]-[K]-[I]-[V]-[V]	8	NO
9	SULFOCYANIN	[C]-[G]-[I]-[AL]-[G]-[H]-[A]-[VAQ]-[SA]-[G]-[M]-[W]	5	1
10	SULFOCYANIN	FNFNGTS	5	1
11	AMICYANIN	[AP]-[G]-[SAT]-[FVY]-[D]-[YF]-[FHIY]-[C]-[RT]-x-[H]-[P]	10	1
12	HALOCYANIN	[C]-[TN]-[P]-[H]-x-[AT]-x-[G]-[M]-x-[G]-[A]	2	NO
13	PSEUDOAZURIN	[K]-[C]-[TA]-[P]-[H]-x-[GAM]-[M]-[GS]-[M]	18	NO
14	AZURIN	[D]-x-[R]-[V]-[LI]-[A]-[HYF]-[T]-x-[VIL]-[VIL]-[G]-[GAS]-[G]	54	NO
15	NITRITE REDUCTASE	[P]-[ST]-[HY]-[VIGA]-[VL]-[FM]-[N]-[G]	235	NO
