# Supplementary material for: Functionally specified protein signatures distinctive for each of the different blue copper proteins
Source: BMC Bioinformatics. 2004 Sep 9;5:127. doi: 10.1186/1471-2105-5-127 (PMC517927; doi:10.1186/1471-2105-5-127)
Supplement: Additional File 1 — Designing protein signatures: Illustrated example Plastocyanin. Plastocyanin sequences of eukaryotic and prokaryotic origin were retrieved from the PDB and SwissProt databases. The eukaryotic sequences were subjected to a ClustalW multiple sequence alignment. Signatures were designed based on the conserved pattern around the active site region [copper binding to four amino acids in plastocyanin]. The same procedure was adopted for plastocyanin sequences of prokaryotic origin. The newly designed signatures were used as queries in the Pattern/peptide match search at the PIR database [Protein Information Resource]. The numbers of plastocyanin sequences retrieved are tabulated in Table 6. The results were compared with the already existing signatures for plastocyanins and the number of sequences that these signatures picked up from the PIR database [data shown in Table 4 & 5]. [file 1471-2105-5-127-S1.doc]

Appendix 1

**Designing protein signature for plastocyanin**

##### Clustal for eukaryotic sequences

**Sequence 1: 1PLB_ 97 aa – Petroselinum crispum**

**Sequence 2: 1AG6__ 99 aa – Spinacia oleracea**

#### Sequence 3: 1KDJ__ 102 aa – Dryopteris crazzirhizoma

**Sequence 4: 1BYO_ 99 aa – Silene pratensis**

>1PLB:

AEVKLGSDDGGLVFSPSSFTVAAGEKITFKNNAGFPHNIVFDEDEVPAGVNAEKISQPEYLNGAGETYEVTLTEKGTYKFYCEPHAGAGMKGEVTVN

>1AG6:

**VEVLLGGDDGSLAFLPGDFSVASGEEIVFKNNAGFPHNVVFDEDEIPSGVDAAKISMSEEDLLNAPGETYKVTLTEKGTYKFYCSPHQGAGMVGKVTVN**

>1KDJ:

**AKVEVGDEVGNFKFYPDSITVSAGEAVEFTLVGETGHNIVFDIPAGAPGTVASELKAASMDENDLLSEDEPSFKAKVSTPGTYTFYCTPHKSANMKGTLTVK**

>1BYO:

AEVLLGSSDGGLAFVPSDLSIASGEKITFKNNAGFPHNDLFDEDEVPAGVDVTKISMPEEDLLNAPGEEYSVTLTEKGTYKFYCAPHAGAGMVGKVTVN


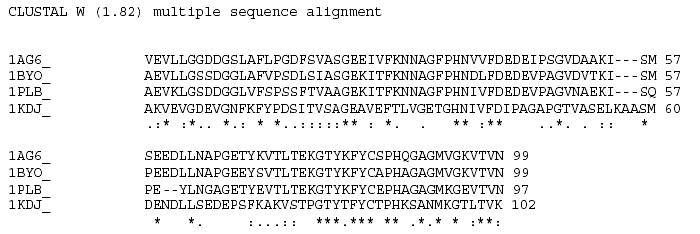


**Eukaryotic plastocyanin protein signature**:

[C]-x-[P]-[H]-x-[GS]-[A]-[GN]-[M]

**Enter the signature in PIR nREF Database**

**[result tabulated in table 5]**

##### Clustal for prokaryotic sequences

**Sequence 1: 1NIN 105 aa- Anabeana variabilis**

**Sequence 2: 1BAW 105 aa- Phormidium laminosum**

**Sequence 3: 1J5C 98 aa - Synechocystosis sp**

**>1NIN**

ETYTVKLGSDKGLLVFEPAKLTIKPGDTVEFLNNKVPPHNVVFDAALNPAKSADLAKSLSHKQLLMSPGQSTSTTFPADAPAGEYTFYCEPHRGAGMVGKITVAG

**>1BAW**

ETFTVKMGADSGLLQFEPANVTVHPGDTVKWVNNKLPPHNILFDDKQVPGASKELADKLSHSQLMFSPGESYEITFSSDFPAGTYTYYCAPHRGAGMVGKITVEG

**>1J5C**

ANATVKMGSDSGALVFEPSTVTIKAGEEVKWVNNKLSPHNIVFAADGVDADTAAKLSHKGLAFAAGESFTSTFTEPGTYTYYCEPHRGAGMVGKVVVD


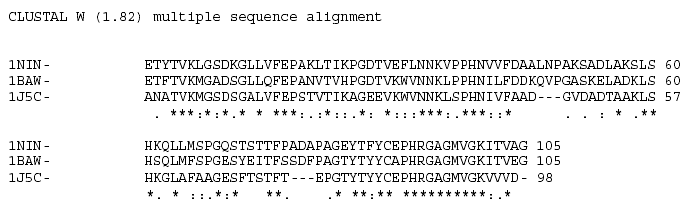


##### Signature for prokaryotic plastocyanin

[Y]-[C]-x-[P]-[H]-[R]-[G]-[A]-[G]-[M]-[V]-[G]

##### Peptide sequence for prokaryotic plastocyanin

PHRGAGMVG

**Enter the signature in PIR nREF Database**

**[result tabulated in table 5]**
